# Case Report: Novel pathogenic variant in *NFIX* in two sisters with Malan syndrome due to germline mosaicism

**DOI:** 10.3389/fgene.2022.1044660

**Published:** 2022-11-09

**Authors:** Elizabeth Langley, Laura S. Farach, Kate Mowrey

**Affiliations:** Department of Pediatrics, Division of Medical Genetics, McGovern Medical School at the University of Texas Health Science Center at Houston (UTHealth Houston) and Children’s Memorial Hermann Hospital, Houston, TX, United States

**Keywords:** NFIX, malan syndrome, germline mosaicism, recurrence risk, genetic counseling, intellectual disability, overgrowth

## Abstract

Malan syndrome is an autosomal dominant disorder caused by pathogenic variants in *NFIX* with less than 100 cases reported thus far. *NFIX* is important for stem cell proliferation, quiescence, and differentiation during development and its protein plays a role in replication, signal transduction, and transcription. As a result of pathogenic variants, symptoms of Malan syndrome include overgrowth, intellectual disability, speech delay, and dysmorphic features. Currently, the recurrence risk for this disorder is indicated at less than 1%, standard for *de novo* autosomal dominant disorders. Herein, we report an additional set of sisters with the same novel pathogenic variant in *NFIX* and clinical features consistent with Malan syndrome providing evidence of germline mosaicism. Considering the rarity of this condition in conjunction with three previous reports of germline mosaicism, it is worthwhile to investigate and re-evaluate the proper recurrence risk for this condition. This discovery would be paramount for family planning and genetic counseling practices in families with affected individuals.

## Introduction

Malan syndrome, also known as Sotos syndrome 2, is an autosomal dominant overgrowth disorder characterized by mild to moderate overgrowth, delayed speech, intellectual disability (ID), and dysmorphic features (Priolo et al., 2018). Malan syndrome is caused by pathogenic variants in nuclear factor I X (*NFIX*) and is located at 19p13.2 [OMIM: 164005] (Malan et al., 2010). Malan syndrome was first described as Sotos-like syndrome, as its presentation is similar to Sotos syndrome that presents with overgrowth, difficulties with learning, cardiac and renal anomalies, seizures, as well as dysmorphic features. However, Sotos syndrome is caused by haploinsufficiency of the *NSD1* gene, rather than pathogenic variants in *NFIX* (Klaasens et al., 2015). *NFIX* is important for stem cell proliferation, quiescence, and differentiation during development and its proteins play a role in replication, signal transduction, and transcription (Malan et al., 2010). Notably, Marshall-Smith syndrome is another rare genetic condition that occurs only from frameshift and splice site variants in exons six to eight of *NFIX* and presents with advanced skeletal maturation, respiratory compromise, and failure to thrive (Klaassens et al., 2015). Malan syndrome was named a distinct entity from both Sotos syndrome and Marshall-Smith syndrome due to its differing clinical presentation and genetic etiology. Since first described in Malan et al., 2010, it is estimated that less than 100 cases of Malan syndrome have been reported.

Although the majority of the prior reports of Malan syndrome are the result of *de novo* pathogenic variants, germline mosaicism was observed in eight individuals from a total of three families (Nimmakayalu et al., 2013; Hancarova et al., 2019; Sihombing et al., 2020). Since rare disorders are underreported, the incidence of Malan syndrome as well as the presence of germline mosaicism may be more prevalent than previously estimated. In general, the recurrence risk for *de novo* variants in most autosomal dominant genetic disorders are estimated to be less than 1% (Rahbari et al., 2016). With the increased availability of next-generation sequencing, it is recognized that there are other factors that can influence the accuracy of this recurrence risk, including the presence of parental somatic mosaicism, mutation rate during embryogenesis, and the gene of interest (Veltman and Brunner, 2012; Samocha et al., 2014; Rahbari et al., 2016). For genetic counseling, it is prudent to highlight the incidence of germline mosaicism in families with Malan syndrome to educate families more precisely on the recurrence risk of this disorder.

Here, we present a case of two full sisters with genetically confirmed Malan syndrome due to a novel frameshift variant in *NFIX*. Both sisters have phenotypes in line with previously described cases and provide further evidence that the incidence of germline mosaicism in Malan syndrome is higher than the standardly quoted less than 1% for other autosomal dominant genetic disorders.

## Case presentation

### Patient 1

**FIGURE 1:**
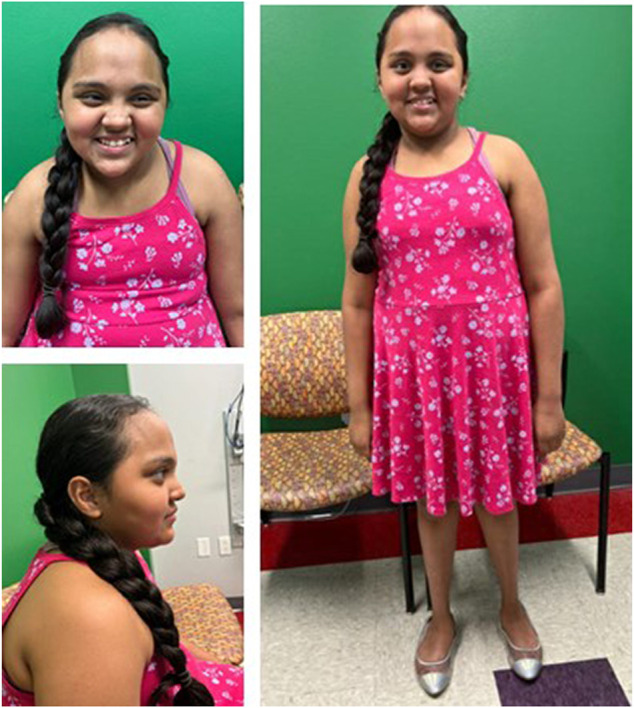
Front (A), profile (B), and full (C) views of patient 1 at 9 years demonstrate overgrowth, macrocephaly, upslanting palpebral fissures, almond shaped eyes, low-set, posteriorly rotated ears, dental crowding, and bitemporal narrowing.

Patient 1 is a 9-year-old Hispanic female born vaginally at 37 weeks gestation to non-consanguineous parents after an uncomplicated pregnancy. After birth, she was in the neonatal intensive care unit for a total of 11 days due to hyperventilation, feeding issues, and jaundice. Motor and speech delays were noted as early as 9 months. Specifically, she pulled to stand at 1 year, cruised at 14 months, and walked at 18 months. At 4 years, she put two words together, and at 9 years, she can speak in two-to-three-word phrases. Due to her global developmental delay, she receives occupational and speech therapy and is enrolled in special education classes. At 4 years, neurology evaluations occurred following a single episode of a febrile seizure. At that time, anti-epileptic drugs were not recommended. At 6 years, she had surgery for bilateral strabismus which was unsuccessful due to optic nerve hypoplasia. A brain MRI at age 7 years demonstrated decreased volume of the bilateral anterior optic pathway. She was 8 years old at her first genetics evaluation and at that time her dysmorphic features included macrocephaly, upslanting palpebral fissures, almond shaped eyes, bitemporal narrowing, flat feet bilaterally low-set, posteriorly rotated ears, a straight border on the inside of her lower legs, and deep, hockey stick hand creases bilaterally (Figures 1A–C). In addition to her overgrowth, she had ID, acquired acanthosis nigricans, and autistic-like features including difficulty communicating, nail biting, and rocking. After evaluation by pediatric neurology, she was diagnosed with autism spectrum disorder of major severity due to persistent deficits in social communication and interaction across multiple contexts, stereotypic behaviors, hyperreactivity to sensory input, and clinically significant impairment in social settings as outlined in the Diagnostic and Statistical Manual 5 (DSM-5). Additionally, these findings were accompanied by language impairment and not better explained by her ID. At her follow up genetics evaluation, she was noted to have an abnormal EEG with continuous generalized slowing, consistent with mild encephalopathy. At this time, anti-epileptics drugs are being considered. At 9 years, her weight is 62 kg (+4 SD) height is 151.6 cm (+2.8 SD) and her head circumference is 58.5 cm (+4.9 SD).

### Patient 2

**FIGURE 2:**
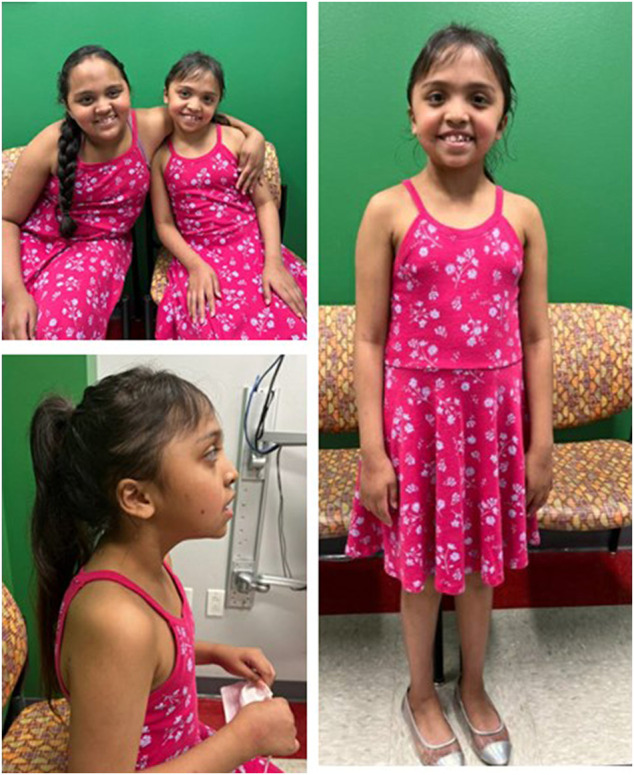
Patients 1 and 2 at ages 9 and 8 years, respectively demonstrate similar facial features (A). Profile (B) and full (C) views of patient 2 at age 8 demonstrating almond-shaped eyes, small, low-set, posteriorly rotated ears, retrognathia, dental crowding, and bi-temporal narrowing.

Patient 2 is an 8-year-old Hispanic female born at term after an uncomplicated pregnancy. She is the full sister of Patient 1. She had delayed speech and did not put 2 words together until 5 years. Her motor milestones were developmentally appropriate. She presents with ID and is enrolled in special education classes. At 6 years, she was diagnosed with paralytic strabismus and did not require surgical intervention. At 7 years, a 23-h EEG was performed and exhibited right focal temporal slow waves without evidence of epileptic or epileptogenic events. Patient 2 was 6 years at her first genetics evaluation and at that time, she could speak in two-to-three-word phrases and received occupational and speech therapy twice a week. Her dysmorphic features included epicanthal folds, hypertelorism, retrognathia, small and low-set, posteriorly rotated ears, and a thick upper lip (Figures 2A–C). At her follow up genetics evaluation, she was noted to have an abnormal EEG due to continuous generalized slowing, consistent with mild encephalopathy, more prominent on the left than the right suggestive of cortical dysfunction. Like Patient 1, anti-epileptic drugs are being considered, but have not been started at this time. At 8 years, her weight is 30.8 kg (+0.77 SD), height is 131.2 cm (+0.56 SD) and her head circumference is 56.8 cm (+3.9 SD).

### Testing

Chromosome Microarray (CMA) was normal for both Patient 1 and Patient 2. Prader-Willi methylation analysis was found to be normal in Patient 1. Patient 2 was identified as a premutation carrier for Fragile X syndrome through Fragile X testing. Lastly, both sisters along with their biological unaffected mother and unaffected father underwent a Quad whole exome sequencing + mitochondrial sequencing (Quad, WES + Mito) analysis. Patient 1 acted as the proband and Patient 2 and their parents were used for segregation analysis. The methodology of the Quad, WES + Mito includes next generation sequencing with copy number variant calling. All variants identified on the testing were confirmed *via* Sanger sequencing. Results revealed that both Patient 1 and Patient 2 had the same novel *de novo* heterozygous pathogenic variant located at c.170_177dupCGAAGGAC (p.E60RfsX8l) [Clinvar ID: 1708174] in *NFIX* [NM_001365902.3]. Specifically, parents were evaluated for this specific genetic change *via* next generation sequencing and were determined to not harbor this pathogenic variant, suggesting that one parent has germline mosaicism. No further haplotyping or additional genetic testing was utilized to elucidate which parent may have germline mosaicism. In addition, a heterozygous known pathogenic variant in *PKP2* [NM_004572.3] located at c.235C>T (p.R79X) [Clinvar ID: 6754] was identified in Patient 1, Patient 2, as well as their mother *via* next generation sequencing leading to an additional diagnosis of arrhythmogenic right ventricular cardiomyopathy (ARVC). To our knowledge, ARVC has not been found to be related to Malan syndrome in the literature. Due to this additional diagnosis, both Patient 1 and Patient 2 were referred to cardiology and have not had any cardiac issues to date.

## Discussion

Here, we present a case of two full sisters with evidence of germline mosaicism leading to Malan syndrome. Of the less than 100 cases presented in the literature, this set of siblings brings the total number of cases of gonadal mosaicism in Malan syndrome to four families and 10 individuals. Given the rarity of this condition and the number of cases of germline mosaicism in Malan syndrome thus far, the recurrence risk may be higher than the standardly quoted less than 1% for this disorder in genetic counseling practices.

While recurrence risk is generally less than 1% for the majority *de novo* genetic diseases, some disorders found to be *de novo* such as Duchenne muscular dystrophy (DMD), Becker muscular dystrophy (BMD), osteogenesis imperfecta type II, Rett syndrome, and Hemophilia A carry a higher recurrence risk of 4–20%, 5–7%, 11%, and 13%, respectively (Byers et al., 1988; Helderman-van den Enden et al., 2009; Mari et al., 2005; Leuer et al., 2001). Generally, germline mosaicism linked to dominantly inherited conditions is rare and is likely due to genes undergoing variations that are not repaired in germ cells (Byers et al., 1988). Like Malan syndrome, osteogenesis imperfecta type II is an autosomal dominant disorder where germline mosaicism has been observed. Byers et al., 1988 determined a recurrence risk of approximately 6% in families with new dominant pathogenic variants due to gonadal mosaicism (Byers et al., 1988). In another study, germline mosaicism was determined by DNA analysis in blood and fibroblast DNA for 13 individuals, while 2 parents were determined to be mosaic based on family structure and having affected children with different partners (Pyott et al., 2014). While the underlying mechanism of recurrence risk in osteogenesis imperfecta type II is not fully understood, this condition provides an example of increased risk for germline mosaicism in an autosomal dominant disorder.

Furthermore, DMD/BMD is an X-linked disorder where germline mosaicism has been well studied and better understood. In Helderman-van den Enden et al., 2009, 19 cases out of the 318 families identified with DMD/BMD with *de novo* pathogenic variants were noted to have to have a second affected male suggesting the presence of germline mosaicism. This study set out to determine the difference between true germline mosaicism and somatic mosaicism. By utilizing haplotyping, the authors were able to determine if the *de novo* pathogenic variant was from the maternal, grandmaternal, and/or grandpaternal X chromosome. The results of the study indicated that in families with no information about the risk haplotype, the recurrence risk was approximately 4.3%. In the families with haplotype information available, the recurrence risk varied from 14 to 20% depending on the risk haplotype that is transmitted (Helderman-van den Enden et al., 2009). This study identified that the recurrence risk for DMD and BMD did not align with the less than 1% risk for *de novo* conditions. As a result, genetic counseling practices have reflected this by educating families with *de novo* cases about the likelihood of having another affected child being higher at 4.3%. Given the increased risk for siblings of Malan syndrome, targeted genetic testing, whether prenatal or postnatally, should be offered. The framework for genetic counseling in DMD and BMD cases may provide a solid foundation to open discussions of how to appropriately modify the recurrence risk for Malan syndrome, so that families can be accurately informed during family planning.

Lastly, regarding clinical features of our patients, they present similarly to other patients with Malan syndrome while harboring a novel pathogenic variant. Although Malan syndrome has variable expressivity, the literature describes core features including global developmental delay, overgrowth, dysmorphic features, strabismus, and ID (Sihombing et al., 2020). This is consistent with the presentation of our two patients.

As previously stated, the pathogenic variant found in both Patient 1 and 2 is novel and not previously reported in the literature to our knowledge. According to ClinVar, 30 pathogenic variants in *NFIX* are associated with Malan syndrome in addition to 11 variants noted to be likely pathogenic (Landrum et al., 2018).

Overall, this sibling set adds another case to the medical literature of germline mosaicism in Malan syndrome and highlights an increasing number of cases of gonadal mosaicism. It appears that germline mosaicism may be more common in Malan syndrome than previously estimated based on the inheritance pattern. This finding may indicate that a higher recurrence risk should be cited for parents of children with Malan syndrome. This topic is essential for genetic counseling and family planning in families of affected individuals.

## Data Availability

The original contributions presented in the study are included in the article/supplementary material, further inquiries can be directed to the corresponding author.
